# Comparing the Efficacy of Individual Approaches and Team-Based Approaches in Solving Clinical Case Vignettes

**DOI:** 10.7759/cureus.47796

**Published:** 2023-10-27

**Authors:** Amita Singh, Amita Kumari, Anita Kumari, Ayesha Juhi, Anup Kumar D Dhanvijay, Mohammed J Pinjar, Himel Mondal, Pratima Gupta

**Affiliations:** 1 Physiology, All India Institute of Medical Sciences, Deoghar, Jharkhand, IND; 2 Physiology, All India Institute of Medical Sciences Deoghar, Jharkhand, IND; 3 Microbiology, All India Institute of Medical Sciences, Deoghar, Jharkhand, IND

**Keywords:** objective question, physiology, clinical problem-solving, decision-making, group size, collaborative learning, medical education, team-based approaches, individual approaches, problem-based learning

## Abstract

Background

Clinical case vignettes are a widely adopted pedagogical approach in medical education. The cases may be presented to students with a closed response option for objectivity. While solving clinical cases has demonstrated its effectiveness in enhancing medical students' clinical reasoning, there is an ongoing debate regarding the most effective approach: individual problem-solving or team-based problem-solving.

Objective

To observe and compare the score obtained from individual clinical problem-solving approaches versus team-based clinical problem-solving approaches.

Methods

After obtaining consent, a total of 100 students were randomly selected for the study. The participants were divided into two groups: an individual approach group (IAG) (n=25) and a team-based approach group (TAG) comprising 25 groups of three students each. Both groups were presented with a set of 10 clinical problems, each requiring a closed-answer response of "yes", "no", or "don't know". The participants' responses were recorded and analyzed to evaluate their problem-solving efficacy.

Results

A total of 25 responses were obtained from 25 students from the IAG group and 25 responses from 25 groups from the TAG group. There was no difference between the score in IAG (7.44±1.12) and TAG (7.52 1.66) p-value=0.58. There was no difference between individual scores in 10 questions between IAG and TAG groups.

Conclusion

The study found no significant score differences between individual and team-based clinical case-solving groups. Hence, for the objective type of case-solving pattern used in this study, a team-based approach may not be necessary. Further research is needed to explore factors for such findings in future studies.

## Introduction

Clinical case vignette-based learning is a widely adopted pedagogical approach in medical education that emphasizes active learning, critical thinking, and collaborative problem-solving. In case-based learning, students are presented with real-world clinical scenarios or problems and are tasked with finding solutions based on their existing knowledge and resources [1, 2]. While case-based learning has demonstrated its effectiveness in enhancing medical students' clinical reasoning and problem-solving skills, there is an ongoing debate regarding the most effective approach: individual problem-solving or team-based learning (TBL) [3].

Individual problem-solving in case-based learning allows students to work independently, relying solely on their own knowledge and problem-solving abilities. In contrast, team-based case-based learning encourages students to work collaboratively in groups, pooling their collective knowledge and skills to arrive at solutions. The combination of team-based learning principles within the framework of case-based learning offers a synergistic approach to medical education [4]. In this integrated approach, students can engage in collaborative problem-solving within their teams but also bring their collective insights to the larger case-based learning group.

However, clinical problem-solving may not always benefit from a team-based approach due to potential challenges such as differing opinions leading to debates, the risk of groupthink, uneven participation, communication issues, decision-making delays, accountability concerns, and compatibility issues within the team. In certain situations, especially those requiring quick and decisive actions or clear individual responsibility, individual problem-solving approaches may be more efficient and effective in ensuring optimal patient care [5, 6]. The choice between individual and team-based approaches should be context-specific and consider the unique demands of each clinical scenario.

The gradual introduction of case-based questions in preclinical subjects for both objective and written examinations represents a forward-thinking pedagogical approach. This shift benefits students by encouraging the practical application of theoretical knowledge, fostering critical thinking, and demonstrating the real-world relevance of their studies. With careful implementation, case-based questions can enhance the overall educational experience and better equip students for their future roles in healthcare or related fields [7, 8]. However, as these examinations are written by individual students, their individual competency is of great importance for academic progression.

Given these considerations, this study aimed to observe and compare the efficacy of individual and team-based problem-solving capability for objective types of questions related to a clinical scenario in physiology among first-year medical students.

## Materials and methods

Type and settings

This cross-sectional study was conducted in a medical school of national importance in the eastern part of India. The study was conducted with first-year medical students studying pre-clinical subjects. The study was conducted on the subject of physiology. This study was approved by the Institutional Ethics Subcommittee of All India Institute of Medical Sciences, Deoghar, Jharkhand, India as an individual project (2023-210-IND-03).

Sample and recruitment

**Figure 1:**
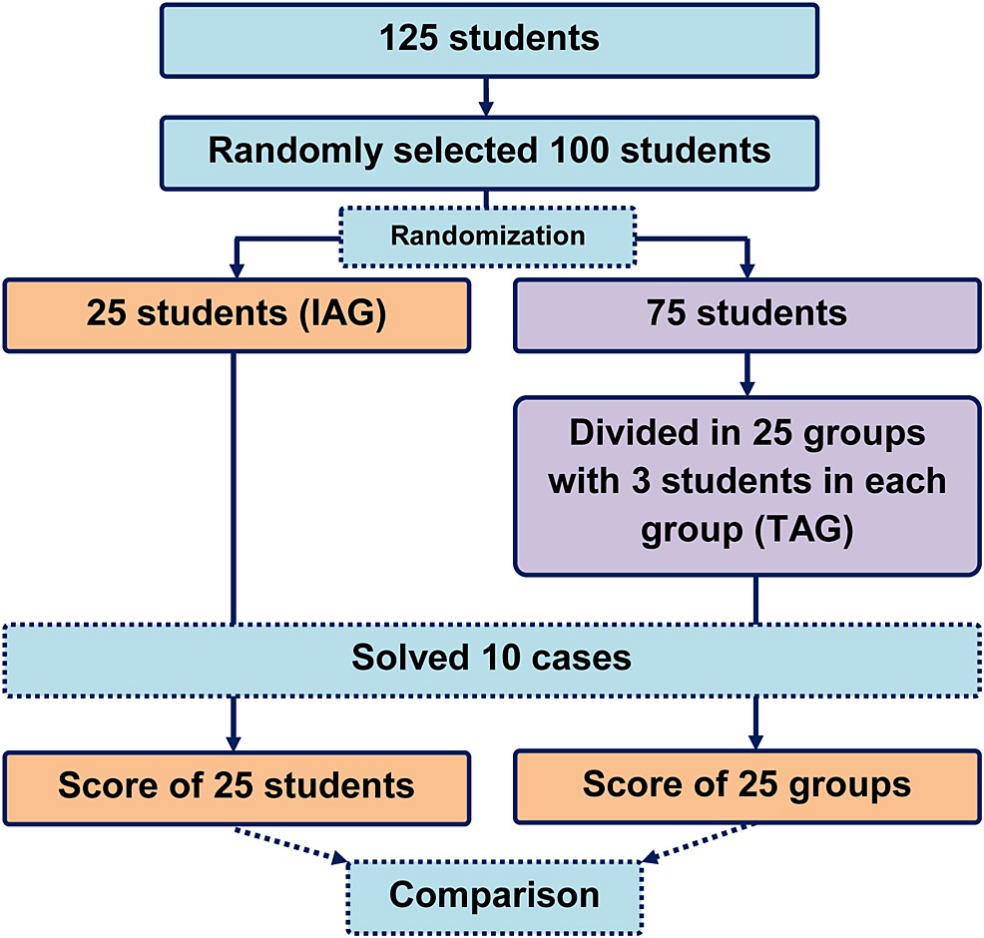
Number of participants and brief study method IAG - individual approach group; TAG - team-based approach group

Total number of students in the medical school is 125. All the students were approached for the study, and 121 students consented to participate in the study. Among the willing students, a randomly selected 100 students were recruited for the study. They were randomly assigned to two groups: the individual approach group (IAG) (n=25) and the team-based approach group (TAG) (n=25; each team contains three students). The process is shown in Figure 1.

Case-based learning questions preparation and validation

**Figure 2:**
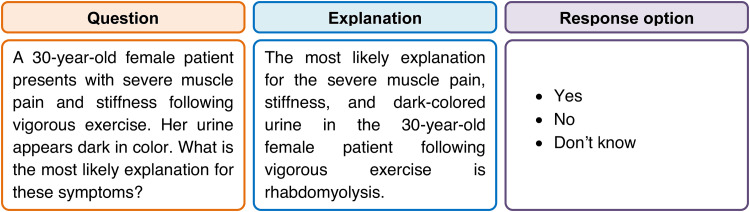
An example of a case, its explanation, and response options

To ensure the validity and reliability of the case-based learning questions, a panel of subject matter experts (n=3) who are experienced in medical education (more than 10 years) in physiology developed the questions and answers. These scenarios and questions are reviewed for face and content validity and clarity by another two experts. Additionally, a pilot test was conducted with a small group (n=10) of second-year undergraduate medical students to find the suitability and comprehension of the questions. Based on feedback and pilot results, necessary adjustments were made to the questions, and finally, 10 questions were prepared and printed on white paper for application. One such question is shown in Figure 2. In this example, the score for the answer yes = 1, don't know = 0, and no = -1.

Data collection

Participants from both the IAG and TAG engage in case-based learning sessions where they work on the provided scenarios and answer associated questions. Their responses were analyzed with pre-defined answer keys. The correct answer was scored 1, the wrong answer was -1, and don't know was 0. The grading system incentivizes correct responses, penalizes incorrect ones, and recognizes instances where students openly admit their lack of knowledge, contributing to a more nuanced understanding of their problem-solving skills.

Statistical analysis

Descriptive statistics were used to summarize the data. The scores obtained by the IAG and TAG groups were compared by the Mann-Whitney U test. We used GraphPad Prism 9.5.0 for statistical analysis, and a p-value <0.05 was considered statistically significant.

## Results

**Figure 3:**
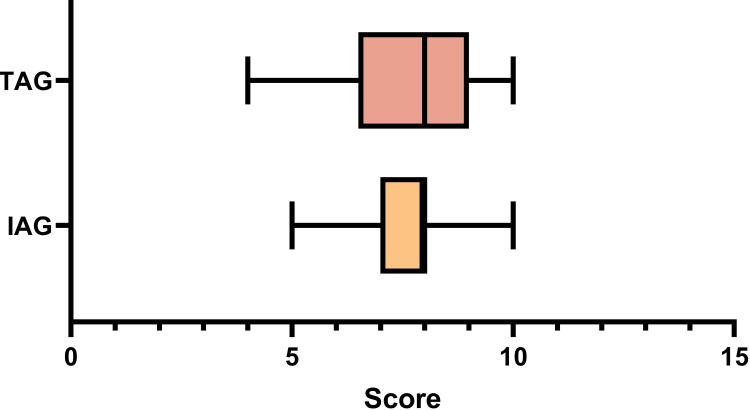
Score of individual approach group (IAG) and team approach group (TAG)

A total of 100 undergraduate medical students in their first year of study participated in this study. Among these 100 students, 25 students comprised the individual group, and 75 students were in the team-based group (each group was composed of three students). The score between the IAG (7.44±1.12) and TAG (7.52 1.66) was similar (p-value = 0.58). This indicates that a student alone and in a group have similar performance in solving objective-type clinical case solving. The score in the box plot is shown in Figure 3.

The case-wise average score is shown in Table 1. There was no difference between the scores in IAG and TAG. This indicates that not only in overall performance, question-wise performance of students in individual or in a group perform equally.

**Table 1:** Problem-wise score in individual approach group (IAG) and team-based approach group (TAG) Q - question

Group	Q1	Q2	Q3	Q4	Q5	Q6	Q7	Q8	Q9	Q10
IAG	1±0	0.76±0.44	0.92±0.28	-0.08±0.86	1±0	0.48±0.59	0.68±0.63	1±0	0.84±0.37	0.92±0.28
TAG	1±0	0.8±0.41	0.92±0.4	-0.44±0.87	0.84±0.55	0.6±0.5	0.92±0.4	0.92±0.4	1±0	0.88±0.44
p-value	>0.99	>0.99	>0.99	0.12	0.49	0.6	0.9	>0.99	0.11	>0.99

## Discussion

TBL is a valuable pedagogical approach with several advantages in case-based learning contexts. TBL fosters collaboration and teamwork skills, which are crucial in healthcare settings where professionals often work in interdisciplinary teams [9]. TBL encourages students to collectively solve complex problems and share diverse perspectives. TBL promotes active engagement by dividing students into teams, requiring them to actively participate in discussions, and holding them accountable for their contributions. This dynamic interaction enhances critical thinking [10].

However, TBL may not be an ideal method for all aspects of case-based learning. The effectiveness of TBL relies on cohesive team dynamics, and if teams experience conflicts or imbalances in participation, it can hinder the learning process. Case-based learning's individual approach may provide students with a more self-directed learning experience, enabling them to tailor their problem-solving strategies to their unique needs [11]. It may build self-confidence and help in memory retention [12]. Therefore, the choice between TBL and individual learning should be made judiciously, considering the specific learning objectives, context, and desired outcomes in medical education [13].

In our study, the lack of a significant difference in scores between the IAG and the TAG could be attributed to several underlying factors. The composition of both groups was relatively homogeneous in terms of academic backgrounds, prior knowledge, and problem-solving skills. Such homogeneity could have led to similar performance outcomes for both groups and individuals. Additionally, the level of complexity of the cases could have played a role. As for objectivity, we used choice-based closed answers. Hence, actual collaborative problem-solving might not take place. The dynamics within each TAG team may have also influenced outcomes, as issues with communication, cooperation, or leadership could have hindered their collective performance. Moreover, some students in the IAG may have taken a highly self-directed approach to problem-solving, compensating for the absence of teamwork [14, 15].

The study's limitations include a relatively small sample size, potential variations in the complexity of case scenarios, and the focus solely on academic performance as an outcome measure. Additionally, the study's cross-sectional design may not capture the long-term impact of case-based learning approaches on students' clinical reasoning and problem-solving abilities.

## Conclusions

In conclusion, this study examined the effectiveness of individual and team-based case-based learning approaches in a medical education context. The results indicate that, within the parameters of this study, there were no significant differences in academic performance scores between the two approaches for objective case-solving in physiology. These findings suggest that the choice between individual and team-based case-based learning methods may not significantly impact immediate academic performance. Further research on other subjects with larger sample sizes may provide deeper insights into the topic.
